# Iron Nanoparticles Derived from Olive Mill Wastewater for Sustainable Soil Remediation

**DOI:** 10.3390/nano16020118

**Published:** 2026-01-15

**Authors:** Mar Gil-Díaz, Carolina Mancho, Rosa Ana Pérez, Juan Alonso, Sergio Diez-Pascual, Beatriz Albero, M. Carmen Lobo

**Affiliations:** 1 Instituto Madrileño de Investigación y Desarrollo Rural, Agrario y Alimentario (IMIDRA), Finca “El Encín”, Autovía A-2, Km 38.2, Alcalá de Henares, 28805 Madrid, Spain; carolina.mancho@madrid.org (C.M.); sergio.diez@madrid.org (S.D.-P.); carmen.lobo@madrid.org (M.C.L.); 2 Instituto Nacional de Investigación y Tecnología Agraria y Alimentaria, Consejo Superior de Investigaciones Científicas (INIA-CSIC), Autovía A-6, Km 7, 28040 Madrid, Spain; perez.rosana@inia.csic.es (R.A.P.); albero@inia.csic.es (B.A.)

**Keywords:** metal(loid)s, PCBs, TCPP, soil pollution, Circular Economy

## Abstract

There is an urgent need to develop sustainable approaches for the remediation of contaminated soil as well as to promote sustainable practices for waste management. Here, we provide the first evaluation of the performance of two types of iron nanoparticles (NA and NH) obtained from olive mill wastewater for the remediation of an acidic multi-contaminated soil, including metal(loid)s, PCBs, and a flame retardant (TCPP). Their efficiency was then compared against that of a commercial nanoscale zero-valent iron (NS) through a one-month microcosm experiment employing two doses of each nanomaterial. The impact of the treatments on key soil physicochemical properties, metal(loid) availability, PCB and TCPP concentrations, and soil phytotoxicity was assessed. All treatments reduced soil acidity. Regarding organic contaminants, bioremediation of TCPP was enhanced by all nanomaterials, particularly NH, whereas NA was the only treatment that significantly reduced PCB concentration under the tested conditions. NS achieved the highest rates of metal(loid) immobilization (63–100%); NH was most beneficial for soil fertility and immobilized As, Ni, and Pb (100, 38, and 53%, respectively), whereas NA was only effective for Pb (21–49%). The low dose of both NA and NH improved the germination index (66 and 61%, respectively), reducing soil phytotoxicity. These results highlight the potential of valorizing olive mill wastewater for soil remediation, thereby contributing to the principles of the Circular Economy.

## 1. Introduction

Human, animal, plant, and environmental health are intertwined, and soil is a vital, but often overlooked, component of this balance, providing ecosystem services such as nutrient cycling, water regulation, biomass production, contaminant filtration, carbon storage, and biodiversity maintenance [1,2,3]. However, several anthropogenic activities in the last century have resulted in substantial soil degradation, thereby diminishing the functional capacity of soil. Accordingly, it is estimated that 33% of soil globally and over 60% of soil in the European Union (EU) are moderately to highly degraded [1,3,4]. In particular, [5] estimated a total annual cost of soil degradation in the range of €40.9–72.7 billion for the EU alone, due to the loss of essential soil services and the impact on human health. Consequently, the EU has put the concept of healthy soils at the core of the European Green Deal to achieve climate neutrality, zero pollution, sustainable food provision, and a resilient environment [6]. Soil contamination is a form of degradation in which harmful substances from human activity reduce soil quality [7]. Mining and industrial activities, agriculture, urban life, transport, and military activities are the main anthropogenic drivers of soil pollution [8]. The contaminants from these activities can be organic or inorganic in nature (metal(loid)s and salts). Polychlorinated biphenyls (PCBs) are a class of man-made persistent organic pollutants with properties of interest (non-flammability, chemical stability, high boiling point, and electrical insulating capacity) for a multitude of industrial and commercial applications [9,10]. They are highly toxic and persistent, which is why they were progressively banned during the 1970s and are included in the Stockholm Convention [9,10]. Organophosphate esters (OPEs) comprise a large class of compounds widely used in industry as flame retardants and plasticizers for a variety of products and applications [11]. One of the most-used OPEs is tris(1-chloropropan-2-yl) phosphate (TCPP), currently registered under the REACH Regulations due to its toxicity and potential carcinogenicity [11,12]. In contrast to organic pollutants, metal(loid)s are non-biodegradable and can remain in the soil matrix for extended periods after their introduction. While total metal(loid) concentrations are commonly referenced in regulatory frameworks, they offer limited insight into actual environmental risk [13]. The concept of bioavailability—referring to the fraction of metal(loid)s that can potentially be absorbed by plant roots and other soil organisms—is now widely adopted [13,14,15]. In this context, there is a growing trend toward implementing remediation strategies aimed at reducing the bioavailability of metal(loid)s in soil [16,17,18,19,20]. Given the multiple sources of contamination and the presence of various types of contaminants in soil, including metal(loid)s and organic compounds, remediation strategies are inherently complex. Therefore, there is an urgent need to develop cost-effective and sustainable approaches for the remediation of soil affected by multi-source contamination.

Iron-based nanoparticles, especially nanoscale zero valent iron (nZVI), have been successfully used to treat soil affected by a variety of contaminants, including metal(loid)s, inorganic anions, halogenated organic contaminants, dyes, antibiotics, and other pharmaceuticals [21,22,23,24,25,26,27]. This nanomaterial has a small particle size (<100 nm in diameter), resulting in a higher specific surface area and greater reactivity than granular iron. nZVI has a core–shell structure that enables multiple contaminant-removal mechanisms. The core of Fe^0^ has marked reductive properties due to its relative low standard potential; it is an electron donor, effective for the reductive precipitation of metal ions and dechlorination of organic solvents [28]. The shell of iron oxides and hydroxides formed as a result of Fe^0^ corrosion enables the sorption and surface complexation of metal(loid)s and organic contaminants [22,29]. Nevertheless, bare nZVI particles have several limitations, including: (i) their tendency to agglomerate, causing a decrease in their reactivity; (ii) nZVI is a powerful but indiscriminate reductant, which decreases its reactive lifetime; and (iii) nZVI can induce toxicity depending on the application conditions [22,23,30,31]. To address these drawbacks, some strategies have been developed to enhance the stability of nZVI without sacrificing its efficiency. These include the use of supported carbon-based materials and the encapsulation of nZVI inside micro- or nanocarbon spheres using an organic compound as the carbonaceous source [32,33,34,35]. This nanomaterial is highly relevant for decontamination applications because the carbon shell has adsorptive properties, and the Fe^0^ of the core has a reductant character. In addition, conventional methods to synthesize nZVI present several limitations, including cost and the need for specialized and costly equipment [36,37]. Therefore, in recent years, research efforts have been channeled into greener approaches, i.e., eco-friendly methods based on the use of plant extracts rich in polyphenols, which provide high antioxidant capacity [36,38]. Thus, the mixture of plant extracts with an iron salt at adequate conditions favors the reduction of Fe^2+^ to Fe^0^. In this regard, [39] demonstrated that carbon-coated nZVI showed superior removal efficiency of Cr(VI) from water samples than bare nZVI. [40] observed that carbon-supported nZVI was less effective than bare nZVI for U removal from mine water samples, although the latter was more susceptible to U desorption at long periods. [41] reported that activated carbon balls supported by nZVI composites showed excellent Cr(VI) removal efficiency in water samples. In addition to maintaining their reducing character and increasing their adsorption capacity, carbon-coated nZVI composites can serve as an activator of persulfate to degrade contaminants [35,42].

The use of a waste product as a carbon source in remediation makes the process more environmentally friendly, aligning with practices of the Circular Economy. In this regard, the EU produces roughly 67% of the world’s olive oil, and the associated waste accounts for nearly 75% of the harvested olive biomass [43,44]. Olive mill wastewater (OMWW) has an acidic pH (between 3 and 5.8) and is composed of water, a mineral fraction, and an organic fraction (4–16%). The latter includes an insoluble part composed of olive pulp, suspended matter, and colloidal particles, and a soluble fraction, which contains polyphenols, tannins, proteins, lipids, organic acids, vitamins, and sugars [44,45,46]. Due to its complex composition, OMWW is not readily biodegradable and has to be properly treated before its reuse in agriculture, discharge into rivers, or application in industrial processes. It is considered one of the most toxic effluents in the agri-food industry, mainly due to its acidity and high phenolic content [44,45]. Currently, OMWW is discarded in the majority of countries, largely due to the lack of uniform legislation for its treatment and reuse [45]. The valorization of this toxic waste into a functional product is a key strategy for promoting Circular Economy practices, as it provides a new function for the waste and reduces resource consumption. Carbon-encapsulated iron nanoparticles derived from OMWW have proved effective for the remediation of metal(loid)s and several drugs in spiked water samples [32,33]. However, the effectiveness of this nanomaterial in polluted soil is unknown. Due to the distinct properties of water and soil, strategies effective in the former may not be suitable for the remediation of the latter. To bridge this knowledge gap, this study assesses, for the first time, the feasibility of two carbon-supported iron nanoparticles synthesized from OMWW for the sustainable remediation of a multi-contaminated soil, while also evaluating their impact on soil physicochemical properties and phytotoxicity. Their efficiency is compared with that of commercial nZVI.

## 2. Materials and Methods

### 2.1. Soil

An industrial soil from Madrid region with concentrations of Ni, Pb, and PCBs exceeding the permissible limits (Orden 2770/2006 and RD 9/2005) was selected for the present study. Soil samples were collected from the surface layer (0–30 cm), air-dried, and sieved (<2 mm). The physicochemical properties of the soil (Table S1) were determined following the Spanish official methodology for soil analysis [47]. Briefly, pH and electrical conductivity (EC) were measured in a 1:2.5 soil-to-water ratio; available P was determined following the Olsen method; organic matter and total N were analyzed using the Walkley–Black and the Kjeldahl methods, respectively; and available Na, Mg, Ca, and K were extracted with ammonium acetate 0.1 N and quantified using a flame atomic absorption spectrometer (FAAS, AA240FS, Varian, Palo Alto, CA, USA). Exchangeable Al was extracted with potassium chloride 1 N, followed by quantitation by inductively coupled plasma optical emission spectroscopy (ICP-OES 5110, Agilent, Santa Clara, CA, USA) [48]. The particle size distribution of the soil was determined using the Robinson Pipette method [49]. The pseudo-total concentration of metal(loid)s was determined after acid digestion of 0.5000 g of soil with a mixture of nitric acid (6 mL, 69%) and hydrochloric acid (2 mL, 37%) in a microwave reaction system (Multiwave Go, Anton Paar GmbH, Graz, Austria). The digestion program consisted of an initial ramp of 10 °C/min to 150 °C (held for 2 min), followed by a second ramp of 3.3 °C/min to 190 °C (held for 45 min). The resulting digestion extracts were filtered through Whatman 541 filter paper and subsequently analyzed by ICP-OES (5110, Agilent). Quality of the analyses was verified by including blanks and a reference soil (SQC001, Sigma-Aldrich, St. Louis, MO, USA). Four-point calibration curves for each element were prepared using the same matrix. Recoveries ranged from 96 to 110%, and the relative standard deviation was below 2%.

The content of seven PCBs (PCB 28, 52, 101, 118, 138, 153, and 180) and TCPP in soil samples was determined after matrix solid phase dispersion with C18 (40–60 µm) using a mixture of ethyl acetate and methanol solution (EtAc:MeOH, 80:20, *v*/*v*). In the extraction procedure, 2 g of soil was spiked with 100 µL of the internal standard mixture and then mixed with 2 g of C18 in a glass mortar. The blended mixture was then transferred to a 20 mL glass column equipped with a cellulose frit at its lower end. The mortar and pestle were rinsed with 5 mL of EtAc:MeOH (80:20, *v*/*v*), which was also transferred to the column, then placed on a multiport vacuum manifold, and the eluates were collected in graduated glass tubes. The collected extracts were evaporated to 1 mL, filtered, and injected into a GC-MS/MS system (Agilent 7890A with a 7000 triple quadrupole mass spectrometer, Waldbronn, Germany), according to the conditions previously described by [50], although without in situ derivatization. In this study, the quantification of TCPP was performed using TCPP-d18. The transitions of TCPP-d18 were 131.0 > 103.0 and 293.0 > 131.0, with corresponding collision energies of 11 and 10 eV, respectively. Stock solutions of PCBs, TCPP, and isotopically labeled internal standards were prepared in ethyl acetate, and stored in amber vials at −20 °C until analysis.

### 2.2. Nanomaterials

Three types of iron nanoparticles were tested: a commercial nanoscale zero valent iron (hereinafter NS) (Nanofer Star from NanoIron, Rajhrad, Czech Republic), and two types of carbon-based iron nanoparticles (hereinafter NH and NA) kindly donated by Calpech (Alicante, Spain). Carbon-based nanoparticles were obtained mixing an iron salt with olive mill wastewater as carbon source.

The nanoparticles were characterized using various methodologies. For the thermogravimetric analysis (TGA), samples were heated from ambient temperature to 500 °C at a constant rate of 10 °C/min in a 30 mL/min flow of N_2_. The organic matter content was determined by oxidation with potassium dichromate. The metal(loid) composition was determined after acid digestion with nitric, hydrochloric, and hydrofluoric acids in a microwave (MultiwaveGo) and quantified using an ICP-OES instrument (Agilent 5110). The size and morphology of the nanoparticles were measured in a JEOL JEM-2100F (Jeol Ltd., Tokyo, Japan) transmission electron microscope (TEM) coupled to an Energy Dispersive X-ray analyzer (INCA Energy 200). The surface area was studied from N_2_ adsorption–desorption isotherms at 77 K in a Micrometrics ASAP 2020 system. Prior to the analysis, the samples were outgassed at 150 and 120 °C for 10 and 2 h, respectively. The X-ray diffraction (XRD) patterns were collected on a Bruker D8 Advance diffractometer with Cu kα1 radiation (1.54060 Å), and a diffraction angle (2θ) from 5° to 90° with a step size 0.04°. Infrared Spectroscopy Analysis was performed by Attenuated Total Reflection Fourier-transform infrared (ATR-FTIR) spectroscopy using a Shimadzu IRAffinity-1S (Kyoto, Japan) apparatus with a diamond ATR accessory, working between 400 and 4000 cm^−1^, 64 scans with a resolution of 8 cm^−1^ and a mirror velocity of 0.6329 cm/s.

### 2.3. Experimental Design

Soil samples (30 g) were weighed in a plastic vial (50 mL) and mixed with NS, NA, and NH at two doses (0.4 and 1%, weight/weight). The treatments were called NS, 2NS, NA, 2NA, NH, and 2NH, respectively. The mixtures were thoroughly homogenized and rewetted with deionized water to reach water holding capacity. Vials were incubated at 28 °C in a climatic chamber in the dark for 7 and 30 days. Six replicates per treatment were included. The pH and redox potential of the incubated soil were monitored throughout the experiment. At each sampling time, three replicates were removed and air-dried for subsequent chemical analysis. To evaluate the capacity of the treatments to immobilize metal(loid)s, as well as the stability of this process, As, Fe, Ni, and Pb were determined using the TCLP test (Toxicity Characteristic Leaching Procedure) (USEPA 1311) at 7 and 30 days. Briefly, 20 mL of sodium acetate buffer (0.1 M, pH 4.93 ± 0.05) was mixed with 1 g of soil and then shaken overnight (18 h). The concentration of metal(loid)s in the extract was then quantified by ICP-OES. After 30 days of incubation, the physicochemical properties of the soil, as well as the concentrations of PCBs and TCPP, were studied following the methodologies previously described.

### 2.4. Soil Phytotoxicity

The phytotoxicity of the soil treated with the iron nanoparticles was determined using a germination test based on the Zucconi test [51,52]. Briefly, twelve watercress (*Lepidium sativum*) seeds were placed in a Petri dish moistened with 6 mL of distilled water (control) or soil extract. After two days of incubation in darkness at 26–27 °C, the percentage of seed germination and root length of seedlings were determined. The germination index (GI) was then calculated as follows: GI (%) = G Ls/Lc, where G is the percentage of germination achieved with respect to the control values, Ls is the mean root length in the soil extracts and Lc is the mean root length in the control.

### 2.5. Statistical Analysis

Data were statistically analyzed using version 30.0.0.0 of the IBM SPSS package for Windows. Analysis of variance (ANOVA) and test of homogeneity of variance (Levene test) were performed. In the case of homogeneity (*p* < 0.05), a post hoc Tukey test was performed to determine the differences among treatments. When homogeneity was absent, the Games-Howell test was used.

## 3. Results and Discussion

### 3.1. Characterization of the Nanoparticles

The three nanomaterials presented distinct properties (Table 1). NS was strongly alkaline, NA was slightly above the neutrality, and NH presented acidic character. NS showed the highest Fe content, followed by NA, whereas NA and NH presented higher content of P, K, S, N, Mn, Na, and Ca than NS. The organic matter content differed among the three nanomaterials, with NH showing the highest percentage (69%), followed by NA (21%), likely due to their organic origin—olive mill wastewater. In contrast, NS did not have an organic fraction and can be considered a mineral material. This finding is consistent with the TGA, which revealed that the weight of NS remained unchanged up to 500 °C, thereby showing good thermal stability (Figure S1a). Conversely, NH showed the highest mass loss (nearly 40%), with a sharp decrease at temperatures above 300 °C. Generally, weight loss between room temperature and 100 °C is attributed to hydration water. From 100 to 300–350 °C, the combustion of the more readily oxidizable compounds, including polysaccharides and aliphatic structures, may occur. At higher temperatures, the breakdown of more aromatic moieties, including lignin and non-hydrolyzable compounds, takes place [53]. This behavior is consistent with the decomposition (pyrolysis) of organic matter [54]. NA showed a higher thermal stability than NH, with only about a 10% reduction in weight. Nitrogen adsorption–desorption isotherms indicated that NS and NH show meso- to macroporosity, while NA had a mesoporous structure, as evidenced by a hysteresis loop at relative pressure (p/p_0_) > 0.4 (Figure S1b). NS showed the highest specific surface area and pore volume values, followed by NA, whereas NH had the lowest values.

**Figure 1:**
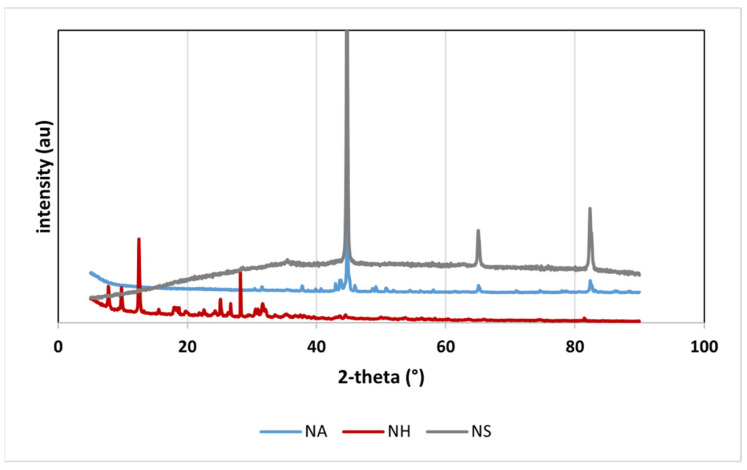
XRD pattern of NA, NH and NS.

According to the XRD pattern of NA (Figure 1), the diffraction peaks at 2θ = 37.72°, 42.95°, 43.57°, 43.82°, 44.72°, 45.09°, 45.93°, 49.17°, 65.05° revealed the presence of iron carbide (ICDD 01-073-9833, 01-089-2722), zero-valent iron (43.57°, 44.72°, 45.09°, 65.05° and 82.36°, ICDD 00-001-1267, 04-014-0264, 00-006-0696), and carbon (31.53° and 45.93°, ICDD 00-046-0944). In addition, some peaks (30.43°, 31.53°, 48.66°, 55.40°, 58.07°, 62.77°, 70.89°, 74.56°, 86.03°, 86.27°, 86.56°) matched with graphite (ICDD 01-089-8488). These results indicate the presence of a core–shell structure in NA, with a core of zero valent iron and a shell made up of various carbon compounds. Regarding NH, the XRD analysis showed that it was mostly amorphous, as no crystalline peaks were observed. It can be due to its high content of organic matter. NS showed diffraction peaks at 2θ = 44.64°, 65.01° and 82.32°, characteristic of α-Fe0 (ICDD 04-013-5208, 04-006-3633), and a weaker peak at 35.48°, which can be attributed to iron oxide (ICDD 00-003-0863, 01-084-2782). These findings confirm that nZVI has a core–shell structure, with a core of Fe^0^ and a thin shell formed by iron oxides, in agreement with previous studies [55,56].

The FTIR spectra showed that NS and NA exhibit no significant peaks, indicating the absence of functional groups. These findings are consistent with previous analyses, as NS consists primarily of Fe and its oxides and hydroxides, while NA is mainly composed of Fe, Fe_3_C, and graphite (layers of hexagonal carbon atoms covalently bonded, with van der Waals forces acting between them). In contrast, NH spectra displayed bands associated with different functional groups, including hydroxyl (a broad absorption band in the range of 3200–3500 cm^−1^, and bands at 1300–1400 cm^−1^, and 600–700 cm^−1^ relative to O–H stretching), carboxylate and carbonyl functional groups (1600–1700 cm^−1^), and aromatic ring (1600–1500, 1200–900 cm^−1^) [57].

**Figure 2:**
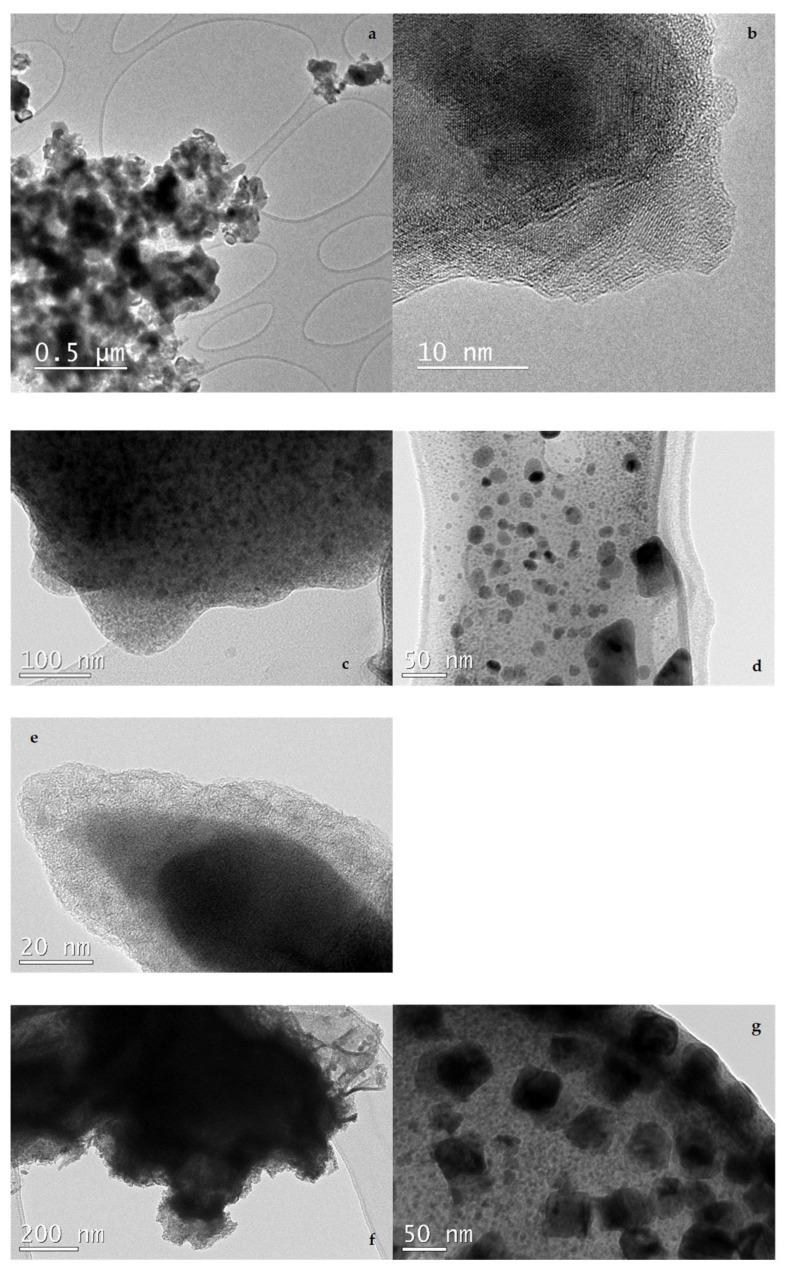
TEM images of NS (**a**,**b**), NA (**c**–**e**) and NH (**f**,**g**).

TEM analysis revealed major differences in the morphology of the three materials (Figure 2). The commercial nZVI used in this study (NS) has been extensively characterized by the supplier (https://nanoiron.cz/en/products/zero-valent-iron-nanoparticles/nanofer-star, accessed on 1 December 2025), as well as by several authors [29,58,59]. TEM analysis confirmed a spherical structure and regular size for NS, in agreement with these previous studies. NA particles appeared partially aggregated and showed different morphologies (spherical, elliptical, and irregular shapes). Most of the particles were surrounded by two thin layers, the first composed of iron oxides and/or Fe_3_C species, and the second mainly made of carbon (Figure 2c–e). This finding is consistent with the XRD results previously mentioned. Analysis of the NH sample showed aggregated particles exhibiting predominantly spherical and cubic morphologies (Figure 2f,g). The latter can be explained by the spinel structures of maghemite or magnetite. In this case, a carbon shell surrounding the iron was not observed; instead, a carbon matrix containing iron-based compound particles dispersed in a pudding-like manner was detected.

### 3.2. Monitoring of pH and Redox Potential

**Figure 3:**
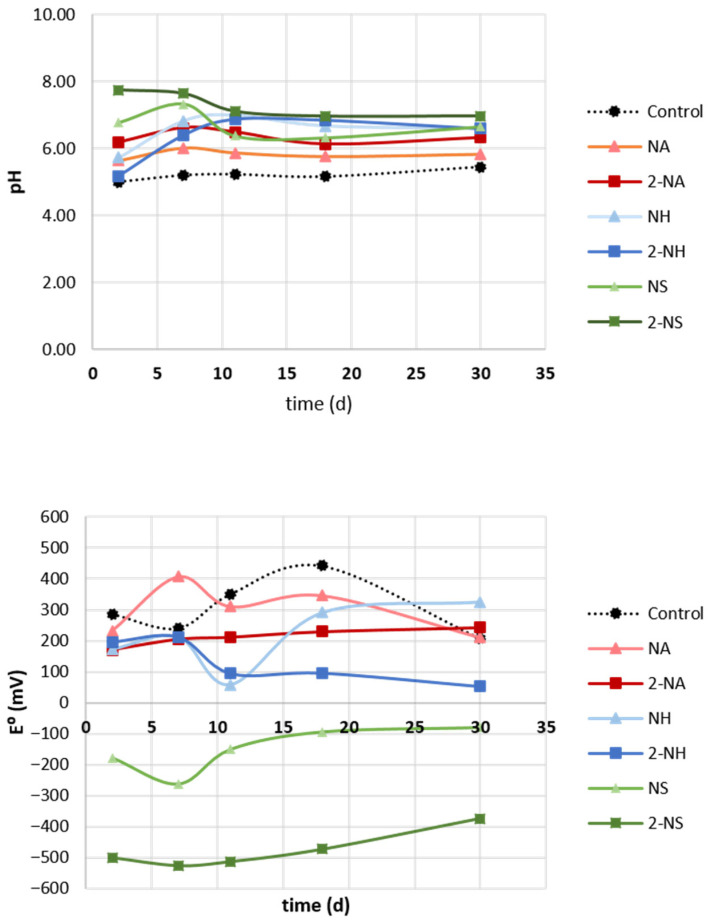
Mean values of soil pH and redox potential (E^0^) throughout the experiment.

During the experiment, soil pH and redox potential were monitored (Figure 3). The values of the redox potential revealed reducing conditions for NS treatments throughout the experiment, especially at the highest dose (2NS, from −500 to −375 mV). In general, the NH and NA treatments showed a lower redox potential than the control, but with positive values. This finding can be explained by the much higher Fe^0^ content in NS compared with NA and NH. Comparison of NA and NH revealed that the 2NH treatment showed a lower redox potential, probably due to the higher content of organic matter rich in phenolic hydroxyl groups, which can accept electrons (Table 1). The three treatments increased soil pH, with NS showing the strongest effect, likely due to its higher alkalinity. In general, the pH value in all three treatments was stable throughout the experiment. The increase in pH is highly relevant for the case of this acidic soil, since pH values more suitable for nutrient availability, microbial activity, and plant development were achieved [60].

### 3.3. Impact on Soil Physicochemical Properties

Table 2 shows the physicochemical properties of the soil after treatments. All three nanoparticle types significantly increased soil pH compared to the control (*p* = 0.001), with NS showing the strongest effect, consistent with the measurements taken throughout the experiment. In parallel, exchangeable Al^3+^ was significantly reduced as a result of the decrease in soil acidity. As previously commented, the agents NS and NA have alkaline pH values, with NS being the strongest (Table 1). In contrast, NH, despite having an acidic pH, contributed to a slight reduction in soil acidity. This effect may be attributed to the characteristics of its organic matter, which has a high cation exchange capacity, enabling the exchange of H^+^ ions and thereby buffering soil pH. Another possible explanation for the increase in soil pH is the consumption of H^+^ ions through microbial reduction reactions [61]. However, this effect should be evaluated over a longer period before NH application on an acidic soil at field scale.

Electrical conductivity also significantly increased by the treatments (*p* = 0.000), especially with 2NH, although the final values were within a normal range for soils. In parallel, NH induced a dose-dependent increase in the exchangeable cations Na^+^ and K^+^. In fact, both types of nanoparticles from OMWW increased the availability of these cations, especially NH, likely due to their composition (Table 1), whereas soil treated with NS and control showed similar values. Olive mill wastewater is comprised mainly of water, organic compounds, and mineral salts in variable percentages depending on the region of origin, olive variety, and the particular oil extraction methodology used. Previous studies found that K^+^, Na^+^, Ca^2+^, Mg^2+^, and PO_4_^3−^ are quantitatively relevant in the mineral fraction of this waste [32,44,45,46]. Under the experimental conditions tested, the Na^+^ levels did not pose a risk to soil quality; however, a higher dose of NH could increase soil salinity, thereby limiting plant development. The presence of Na^+^ in the wastewater can be associated with the use of NaCl during the process of olive oil extraction [62]. To limit the content of Na^+^ in NH, the synthetic process could be adapted, for instance, by including additional washing steps. In contrast, the increase in soil K^+^ content in NH treatments can be considered a positive effect since it is an essential nutrient for plants. NA also induced a significant increase in soil K^+^ content, but only at the highest dose. No significant effects were observed for the exchangeable fractions of Ca^2+^ and Mg^2+^. The latter was slightly increased in the treatments with nanoparticles obtained from OMWW, although significant differences were found only for the 2NA treatment (*p* = 0.004). Moreover, soil enrichment with K^+^ may reduce Mg^2+^ and Ca^2+^ availability due to antagonistic effects [44]. The NS treatments did not affect exchangeable cations.

Regarding the soil organic matter content, treatment with 2NH resulted in a significant increase in this parameter, compared Control, NA, NS and 2NS, likely explained by the characteristics of NH (Table 1). However, from the perspective of soil science, the increase is considered slight. With respect to available P, neither NA nor NH had a significant impact on this parameter, and values were similar to those observed in the control. In contrast, NS reduced available P in a dose-dependent manner (a reduction of 58% for NS and 78% for 2NS). This finding reveals the capacity of nZVI to immobilize phosphate, as concluded by previous studies [63,64,65]. This capacity must be considered before applying NS, as it can lead to reduced soil fertility. A comparison of the performance of the three nanomaterials shows that NH had the most beneficial impact on soil fertility.

### 3.4. Impact on Metal(loid) Availability

**Figure 4:**
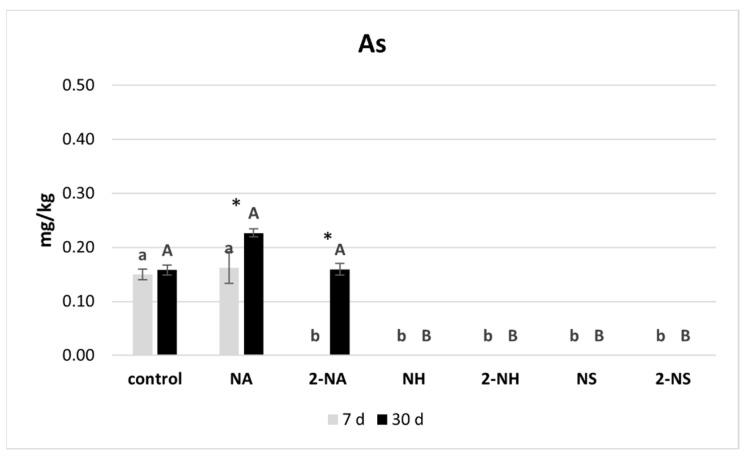

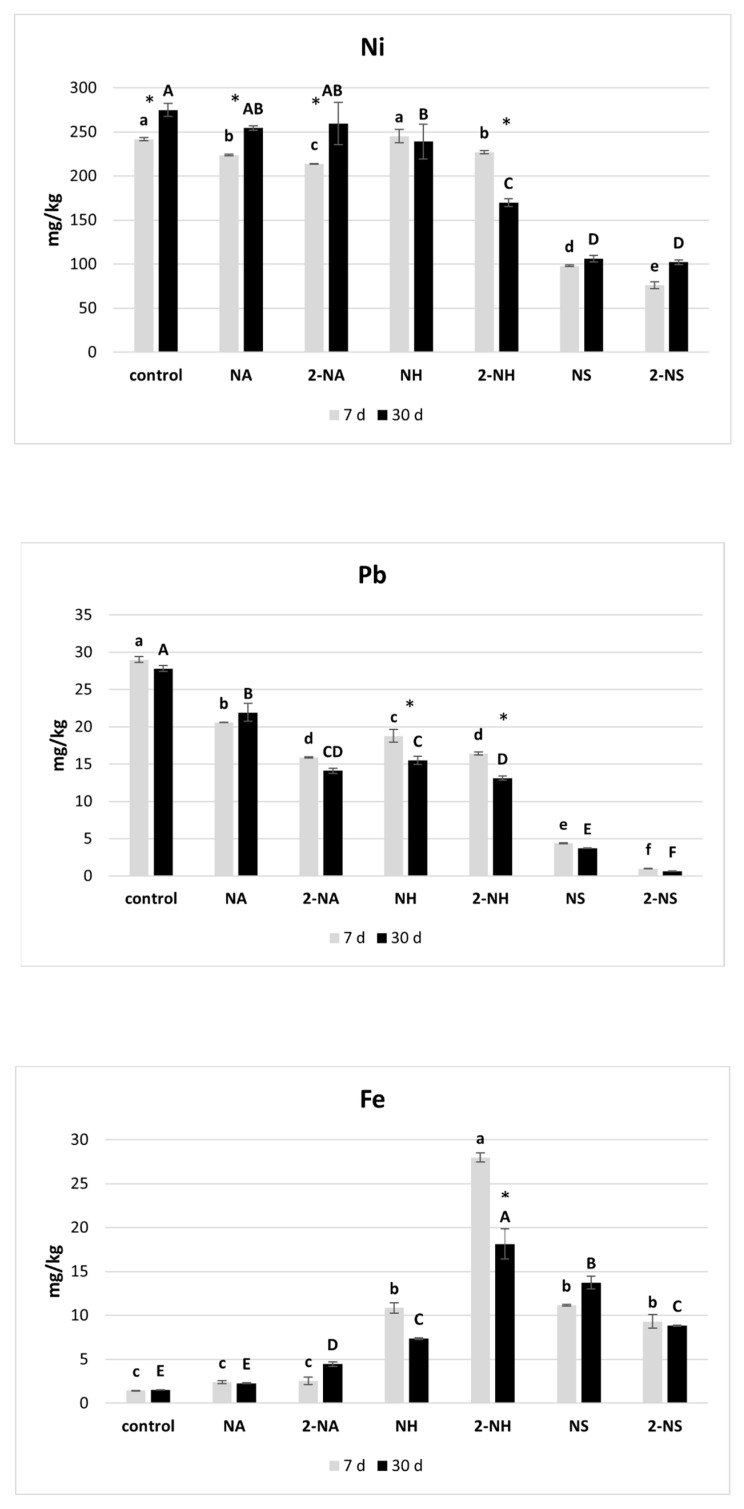
Mean concentrations and standard deviations (mg/kg) of metal(loid)s in TCLP extracts at 7 and 30 days for the different treatments. Bars followed with the same letter do not significantly differ (*p* < 0.05). Lowercase letters indicate significant differences at the 7-day sampling time, while uppercase letters indicate significant differences at 30 days. For each treatment, * indicates significant differences between both sampling times (*p* < 0.05).

The results of the TCLP test for the soil under the different treatments are shown in Figure 4. NH and NS were effective for As immobilization (*p* < 0.001), regardless of the dose, and this parameter remained stable throughout the experiment. In contrast, NA reduced As availability only at the highest dose (2NA), but the immobilization was reversible. In fact, in the 30-day sampling, As availability was similar to that of the control. In the case of Ni, NS showed the greatest immobilization capacity, with reductions of over 60% (*p* < 0.001). In the short term (7 days), the 2NS treatment was more effective at immobilizing Ni than the low dose (NS), but after 30 days, the two doses gave comparable results. Thus, the lowest dose of NS would be enough to immobilize Ni. NH showed enhanced Ni immobilization capacity with increased dose and contact time, with the 2NH achieving a 38% reduction in Ni after 30 days. In contrast, NA treatments showed lower effectiveness, and reduced Ni availability only at the first sampling time (7.5 and 12% for NA and 2NA, respectively). However, the immobilization of Ni with NA did not remain stable after a month. Regarding Pb, NS showed the highest immobilization capacity, while NH achieved only slightly better immobilization rates than NA (*p* < 0.001). The reduction in Pb availability with NS was dose-dependent, reaching 97% in the 2NS treatment, and remained stable throughout the experiment at the two doses tested. For the NH treatments, a longer contact time significantly increased the effectiveness for Pb immobilization, i.e., NH reduced Pb-TCLP from 35 to 44% after 7 and 30 days, respectively (*p* = 0.005), whereas 2NH achieved a decrease from 44 to 53% (*p* < 0.001). NA showed dose-dependent effectiveness for Pb immobilization, with 2NA achieving a 49% decrease in leachability after 30 days—a higher reduction than those observed for As and Ni.

In summary, the present study demonstrates, for the first time, the capacity of nanoparticles obtained from olive mill wastewater to reduce metal(loid) mobility in soil under the experimental conditions tested. However, these nanomaterials were less effective than commercial nZVI nanoparticles. Firstly, NS induced a higher increase in soil pH than carbon-based nanoparticles, which favors the retention of metal cations such as Pb^2+^ and Ni^2+^ [26,27,66,67]. In this regard, ref. [68] reported a drastic decrease in Ni availability at soil pH above 6.5. Furthermore, the Fe content and the characteristics of the organic matter of the nanomaterials play a key role in metal(loid) immobilization capacity [66]. Although elucidation of the interaction mechanism is beyond the scope of this manuscript, we propose that the carbonaceous matrix, as well as the iron oxides, can favor adsorption and complexation reactions, whereas the iron core may be responsible for reductions. In this regard, the standard reduction potentials of Ni^2+^ and Pb^2+^ (E^0^ = −0.24 V, −0.14 V, respectively) were slightly more positive than that of Fe (E^0^ = −0.41 V), indicating that reduction can occur simultaneously with sorption [26,27,28]. Likely, in NH with a 12% of Fe and a high organic matter content (69%, Table 1), complexation with hydroxyl and carboxyl surface functional groups and exchangeable processes of Ni/Pb can predominate versus redox reduction. FTIR analysis evidenced the presence of various oxygen functional groups in NH (Figure S1c). In fact, ref. [69] concluded that organic matter plays a crucial role in controlling Ni availability in soil, particularly during anoxic–oxic alteration. In contrast, such interactions did not occur in NA due to its distinct composition, characterized by iron surrounded by iron carbide and graphite, which exhibit low reactivity toward Ni and Pb.

The mobility of As in soil is strongly associated with the clay fraction, Fe oxides and hydroxides, and organic matter [16,66]. The effect of organic matter on the As bioavailability presents controversial results [16,66]. In this study, the addition of NA (21% of organic matter, 40% Fe, Table 1) was ineffective for As immobilization whereas NH (69% organic matter, 12% Fe), significantly reduced As availability. As previously commented, after seven days, the highest dose of NA (2NA treatment) significantly reduced As availability (*p* < 0.001) but the immobilization did not remain stable. The retention was likely due to interaction with iron since the shell of graphite has poor affinity to As. Further studies are necessary to understand the mechanism and improve the stabilization.

Therefore, the effectiveness depended on the specific metal(loid) and the nanoparticle properties, with NH outperforming NA in As and Ni immobilization, whereas similar rates were observed for Pb with NH treatments and 2NA. In contrast, NA showed a greater capacity in the short term to immobilize Ni than NH, although this immobilization was not stable after 30 days. Accordingly, Ni may interact with NA by reversible adsorption on the carbon-graphite shell. Therefore, this type of material could be used to treat polluted waters and subsequently be removed after the adsorption process. In this context, a previous study involving water samples artificially polluted with Zn, Cd, Ni, Cu, and Cr and treated with iron nanoparticles derived from OMWW found varying degrees of remediation effectiveness depending on the synthesis procedures, with the best achieved using thermally activated nanoparticles [32]. However, water is a simpler matrix than soil, and results obtained in aqueous systems cannot always be extrapolated to soil environments. To the best of our knowledge, limited data are available on the capacity of nanomaterials obtained from OMWW to remediate soil contaminated with metal(loid)s. The use of NH for soil remediation is highly promising, as it effectively immobilized metal(loid)s, enhanced soil fertility, and simultaneously valorized a toxic waste, in line with the principles of the Circular Economy. However, further research is necessary to determine the stability of the metal(loid) immobilization over time. Regarding nZVI particles, our results are consistent with the findings of previous studies that have demonstrated their effectiveness at immobilizing As, Ni, and Pb in polluted soils [17,26,27,67,70,71].

We also evaluated the impact of the three types of nanoparticles on Fe availability since they all contain this element, and little data exist regarding the potential leaching of this type of particles obtained from OMWW. NH had the greatest impact, showing a dose-dependent increase in soil Fe availability (*p* < 0.001), followed by NS (*p* < 0.001). In contrast, NA showed little effect on this parameter under the experimental conditions tested. These results indicate that NH has a higher soluble Fe content than NA despite having a lower total Fe content. Thus, Fe dissolution was limited in the NA treatment, likely due to the graphite shell. These findings demonstrate that the contribution of Fe from the studied nanomaterials to the soil is influenced not only by their total Fe content but also by the specific solubility of this metal within each nanomaterial matrix. Despite its lower Fe content, NH showed greater leaching, probably due to its carbon surface layer is more labile than that of NA, which promotes iron dissolution. Regarding the commercial nZVI, it also led to a significant increase in soil Fe availability at both doses, and this increase remained stable throughout the experiment. The acidic soil pH likely favored the leaching of Fe, as previously reported [63,64]. The increase in soluble Fe in the soil is relevant because this metal is an essential micronutrient for plant growth, which in turn contributes to soil recovery.

### 3.5. Impact on Organic Pollutants: PCBs and TCPP

**Figure 5:**
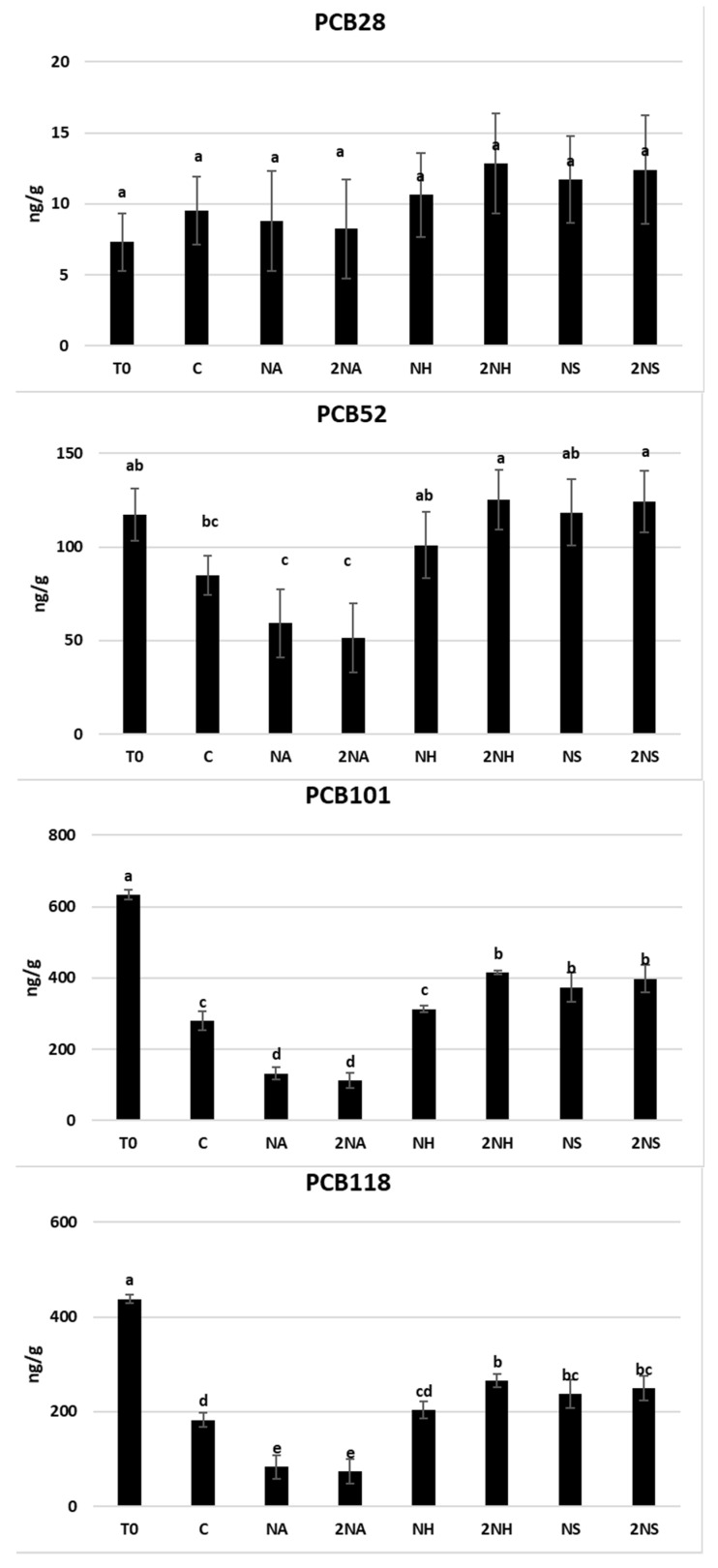

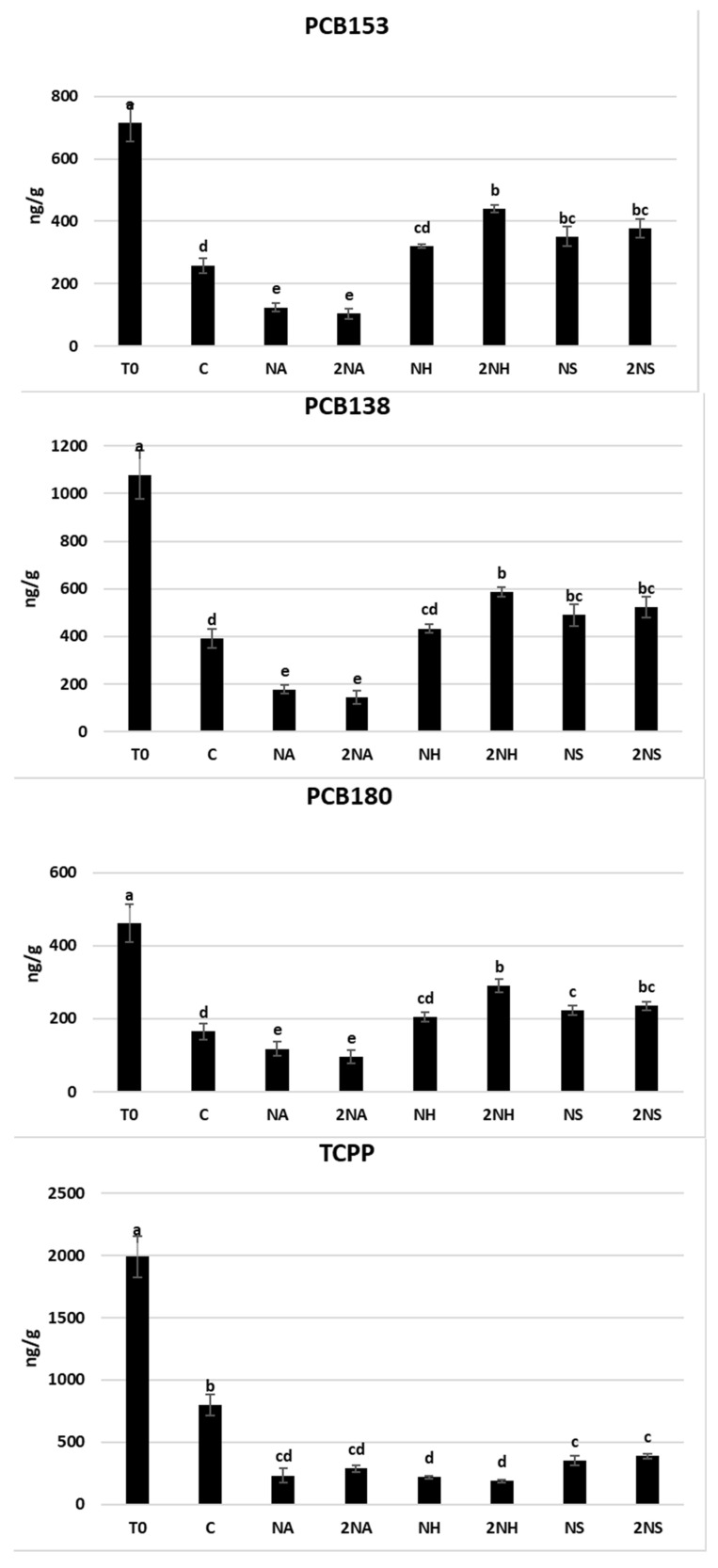
Mean concentrations and standard deviations of PCBs and TCPP (ng/g) in soil samples at the different treatments. Bars followed with the same letter do not significantly differ (*p* < 0.05). T0 represents soil before incubation experiment; C represents control treatment.

The mean content of PCBs and TCPP in the soil after the treatments is shown in Figure 5. In general, NA was the most efficient treatment at removing PCBs from the soil, whereas NH and NS did not reduce these contaminants under the experimental conditions tested. Both doses of NA led to a significant decrease in PCB content (*p* < 0.001), except for PCB28 and PCB52. A reduction ranging from 29 to 63% compared with the control was observed, reaching the highest reduction for PCB138. The mechanism of interaction between NA and PCB molecules was likely based on adsorption on the carbonaceous shell of the nanomaterial. In this regard, a previous study addressing water samples artificially contaminated with metronidazole, sulfamethoxazole, and diclofenac concluded that carbon-encapsulated Fe nanoparticles from OMWW are an effective and fast adsorbent for these contaminants [33]. Our results demonstrate, for the first time, the functional performance of this kind of nanomaterial obtained from OMWW to remediate soil polluted with PCBs. It should be noted that the control treatment showed a lower concentration of PCB101, PCB118, PCB153, PCB138, and PCB180 compared to the original sample (T0), with reductions in the range of 56–64%. These reductions may be explained by bioremediation processes favored by the incubation conditions. In contrast, soils treated with NS, 2NS, and 2NH showed higher concentrations of these PCBs than the control (in the range of 25–76%). One possible explanation is that, under the experimental conditions tested, these treatments may have partially inhibited soil biological activity, thereby limiting PCB degradation. In the case of 2NH treatment, the increase in exchangeable Na^+^ may have negatively affected the microbial population. Regarding NS, previous studies have shown that higher doses of nZVI are effective for the remediation of soils contaminated with PCBs, suggesting that the concentration and application conditions, as well as soil properties, play a critical role in the efficacy of the treatment [56,72]. Thus, NS and NH would not be effective for PCB removal in soil under the experimental conditions tested.

Regarding TCPP, all three nanoparticle treatments reduced its concentration regardless of the dose applied, with NH being the most effective, achieving a reduction of 73 and 77% at the low and high dose, respectively. To the best of our knowledge, this is the first study to evaluate the efficiency of iron nanoparticles at removing TCPP from environmental matrices. Developing strategies to remove TCPP from soil is of great importance due to its ubiquity, persistence, toxicity, and potential for diffusion into other environmental compartments [11,73]. In addition to the adsorption of TCPP by the nanoparticles, we also observed bioremediation, as evidenced by the 60% decrease in TCPP levels in the control soil compared to the original sample (T0). In this context, recent laboratory studies have identified bacterial strains capable of degrading TCPP and other chlorinated organophosphate esters, primarily through the hydrolysis of phosphate ester bonds [74,75,76,77].

Both carbon-based nanoparticles exhibited distinct behaviors toward organic contaminants. NA was effective in removing PCBs and TCPP, whereas NH only achieved a reduction in TCPP. This contrasting performance may be attributed to differences in both contaminant characteristics and nanomaterial properties. Multiple mechanisms are likely involved, including adsorption phenomena and biological and/or chemical degradation. Experimental conditions—such as dose, temperature, contact time, contaminant concentration, and the presence of mixed contaminants—also play a critical role in determining the dominant process. PCB molecules exhibit higher hydrophobicity compared to the organophosphate ester TCPP (log Kow for PCB > 6 vs. log Kow for TCPP = 2.59 [78,79]. According to characterization analysis, NA consists of a zero-valent iron core surrounded by two thin layers: the first composed of iron oxides and/or Fe_3_C species, and the second primarily of partially graphitized carbon, which imparts hydrophobicity. The complexity of this carbonaceous matrix may be critical for its ability to interact with various pollutants. In contrast, NH is an amorphous matrix rich in organic matter (69%), with higher polarity than NA. Consequently, NA is expected to exhibit greater adsorption capacity for PCB molecules than NH, mainly due to hydrophobic interactions. Additionally, NA possesses a higher specific surface area than NH, further increasing its effectiveness. In line with these findings, [80] reported that activated carbons efficiently adsorb 2-PCB in pure water under static conditions, driven primarily by the low solubility of PCB and the hydrophobic nature of activated carbon. Similarly, [81] demonstrated that a composite of porous carbon and nanoscale zero-valent iron achieved efficient PCB dechlorination in aqueous media at room temperature, although the nanomaterial dosage was substantially higher than that used in the present study. Regarding TCPP, all three types of nanoparticles were effective in reducing its concentration. A plausible common interaction mechanism may involve Lewis acid–base interactions, where Fe from the nanomaterial acts as a Lewis acid and TCPP as a weak Lewis base. Supporting this, previous experiments in aqueous media demonstrated that Fe-modified biochar effectively adsorbed TCPP from water through mechanisms including pore-filling, hydrophobic interactions, and Lewis acid–base interactions [82].

The commercial nZVI (NS) exhibited lower efficiency in removing organic contaminants compared to OMWW-derived nanoparticles. Soil amendment with NA emerges as a promising strategy for the removal of PCBs and TCPP, while NH is effective only for TCPP adsorption. Given that NA and NH are obtained from a toxic waste product, their repurposing implies a valorization process aligned with Circular Economy principles. Furthermore, designing a bioremediation strategy assisted by NA nanoparticles could be an efficient approach to reduce organic pollution in the soil tested herein.

### 3.6. Impact on Soil Phytotoxicity

**Figure 6:**
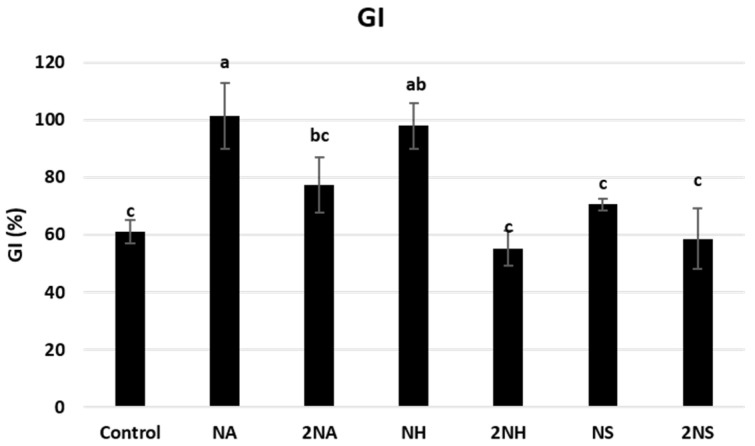
Mean values and standard deviations of germination index (%) of watercress for the soils at the different treatments. Bars with the same letter do not significantly differ (*p* < 0.05).

The impact of the treatments on soil phytotoxicity was evaluated through a germination test using watercress seeds. The results showed that the low dose of NA and NH led to a significant reduction in soil phytotoxicity (*p* < 0.001), whereas this effect was not observed at the high dose (Figure 6). The NH treatment led to a decrease in soil acidity, as well as the leachability of As, Ni, and Pb, and the TCPP content, thereby, NH treatment could induce a reduction in the soil toxicity. In contrast, the 2NH treatment, which generally showed better immobilization results than NH and similar effectiveness for TCPP, did not lead to a decrease in toxicity, probably due to the increase in exchangeable Na^+^ (from 11 mg/kg in untreated polluted soil to 307 mg/kg, Table 2), which may offset these effects. High Na^+^ levels increase the osmotic potential of soil solution, making it harder for plants to absorb water. The NS treatments did not impact soil phytotoxicity, as shown by the germination of watercress seeds. It should be taken into account that the germination tests show some variability, and minor changes in phytotoxicity may go undetected. Overall, this study serves as a starting point: soil treatment with NA or NH has the capacity to reduce soil toxicity and favor seed germination depending on the dose used.

Our results reveal that none of the nanomaterials showed superior performance in all the parameters tested; instead, each exhibited its own strengths: NS excelled in metal(loid) immobilization, NA was the most effective at reducing PCB content, and NH, although less efficient than NS at reducing metal(loid) availability, improved soil fertility and reduced soil phytotoxicity. Therefore, combining these materials emerges as a promising strategy to capitalize on their complementary properties, while simultaneously contributing to waste valorization—a key pillar of the Circular Economy that promotes waste reduction and minimizes resource consumption.

## 4. Conclusions

The present study reveals, for the first time, the capacity of two types of iron nanoparticles (NA and NH), derived from olive mill wastewater, to remediate a multi-contaminated acidic soil. After one month, both waste-derived nanomaterials had reduced soil acidity and phytotoxicity (at the low dose tested), with NH improving soil fertility. The highest rates of metal(loid) immobilization were achieved with commercial nZVI (NS) (63–100%), followed by NH (38–100%), whereas NA showed the least effectiveness. Regarding organic contaminants, bioremediation was enhanced by the use of all the nanomaterials for TCPP while NA was the only treatment effective to reduce the concentration of PCBs (29–63%). Therefore, for the remediation of this site, it would be worthwhile exploring a bioremediation strategy involving a combination of NA and NH nanomaterials to address both metal(loid) and organic contamination while enhancing soil fertility. These findings highlight the potential for valorizing olive mill wastewater as a resource for sustainable soil remediation, thereby contributing to the principles of the Circular Economy. Nevertheless, further research is required to assess the long-term impact of these novel strategies before field-scale application. Additionally, integrating these materials with other remediation approaches should be explored to improve overall effectiveness and enhance soil properties.

## Figures and Tables

**Table 1:** Main physicochemical characteristics of nanoparticles tested.

Parameter	NS	NA	NH
pH	11.0	7.93	5.06
Specific surface area (m^2^/g)	28.2	24.7	15.7
Pore volume (cm^3^/g)	0.143	0.079	0.064
Organic matter (%)	-	21	69
Fe (%)	93	40	12
P (%)	0.08	3.2	3.5
S (%)	0.11	5.8	2.4
Na (g/kg)	0.08	7.4	15
K (g/kg)	0.37	16	13
Ca (g/kg)	0.17	0.90	0.98
Mg (g/kg)	0.30	2.6	0.59
Mn (g/kg)	0.73	2.2	1.4
As (mg/kg)	37	-	-
Cu (mg/kg)	1880	49	19
Ni (mg/kg)	320	500	46
Pb (mg/kg)	-	-	-
Sb (mg/kg)	63	25	5.5
Zn (mg/kg)	470	830	650

**Table 2:** Impact of treatments on physicochemical soil properties (mean values and standard deviation (SD)). Columns with different letters indicate significant differences (*p* < 0.05), and *p*-values are provided in the first column.

Parameter		Control	NA	2NA	NH	2NH	NS	2NS
pH	mean	5.80 d	6.08 c	6.35 b	6.21 bc	6.23 bc	6.80 a	6.76 a
*p* = 0.001	SD	0.05	0.03	0.05	0.04	0.07	0.07	0.03
EC (dS/m)	mean	0.70 c	0.83 b	0.82 b	0.83 b	1.21 a	0.82 b	0.82 b
*p* = 0.000	SD	0.02	0.06	0.05	0.06	0.05	0.04	0.02
N (%)	mean	0.066 a	0.066 a	0.068 a	0.065 a	0.068 a	0.073 a	0.067 a
*p* = 0.777	SD	0.010	0.004	0.003	0.003	0.004	0.010	0.006
OM (%)	mean	0.85 b	0.93 b	1.01 ab	1.01 ab	1.20 a	0.85 b	0.81 b
*p* = 0.000	SD	0.06	0.050	0.101	0.079	0.068	0.079	0.085
P (mg/kg)	mean	36 a	39 a	45 a	48 a	44 a	15 b	8 b
*p* = 0.000	SD	6	4	8	6	9	3	3
Na (mg/kg)	mean	11 e	43 d	79 c	126 b	307 a	20 e	20 e
*p* = 0.000	SD	3	3	2	10	11	10	3
K (mg/kg)	mean	157 de	173 d	210 c	276 b	493 a	142 e	143 e
*p* = 0.000	SD	12	7	7	12	16	9	7
Ca (mg/kg)	mean	573 a	580 a	588 a	582 a	582 a	577 a	546 a
*p* = 0.797	SD	35	36	22	33	40	25	41
Mg (mg/kg)	mean	75 bc	86 ab	97 a	81 ab	88 ab	76 bc	67 c
*p* = 0.004	SD	5	7	7	4	3	13	9
Al (mg/kg)	mean	2.2 a	0.39 b	0.25 b	0.47 b	0.38 b	0.37 b	0.28 b
*p* = 0.000	SD	0.60	0.03	0.05	0.08	0.07	0.03	0.03

## Data Availability

The raw data supporting the conclusions of this article will be made available by the authors on request.

## Supplementary Materials

The following supporting information can be downloaded at: https://www.mdpi.com/article/10.3390/nano16020118/s1, Table S1. Physicochemical properties of the polluted soil. Figure S1. Thermogravimetric analysis in N2 for NA, NH and NS (a). Nitrogen adsorption-desorption isotherms of NA, NH and NS at 77 K (b). FTIR spectra of NA, NH and NS (c).
