# Supplementary material for: Iron Nanoparticles Derived from Olive Mill Wastewater for Sustainable Soil Remediation
Source: Nanomaterials (Basel). 2026 Jan 15;16(2):118. doi: 10.3390/nano16020118 (PMC12844491; doi:10.3390/nano16020118)
Supplement: Supplementary file 1 [file nanomaterials-16-00118-s001.zip › nanomaterials-4048321-supplementary.pdf]

Supplementary Material of:

Iron Nanoparticles derived from Olive Mill Wastewater for Sustainable Soil Remediation

Mar Gil-Díaz*[1], Carolina Mancho[1], Rosa Ana Pérez[2], Juan Alonso[1], Sergio Diez-Pascual[1], Beatriz Albero[2], M. Carmen Lobo[1]

[1]IMIDRA, Finca "El Encín". Autovía A-2, km 38.2. 28805 Alcalá de Henares (Madrid), (Spain)

[2]INIA-CSIC, Autovía A-6, km 7, 28040 Madrid (Spain)

***Corresponding author:** mar.gil.diaz@madrid.org

Table S1. Physicochemical properties of the polluted soil.

Parameter	Mean value
pH	5.33
EC (dS/m)	0.85
N (%)	0.065
OM (%)	0.87
P (mg/kg)	39
K (mg/kg)	145
Na (mg/kg)	19.0
Ca (mg/kg)	680
Mg (mg/kg)	110
Al (mg/kg)	5.6
As (mg/kg)	6.9
Cd (mg/kg)	<LD
Cr (mg/kg)	22.9
Cu (mg/kg)	17.5
Ni (mg/kg)	480
Pb (mg/kg)	160
∑PCBs (ng/g)	3400
TCPP (ng/g)	2000
Sand (%)	10.0
Silt (%)	67.7
Clay (%)	22.3

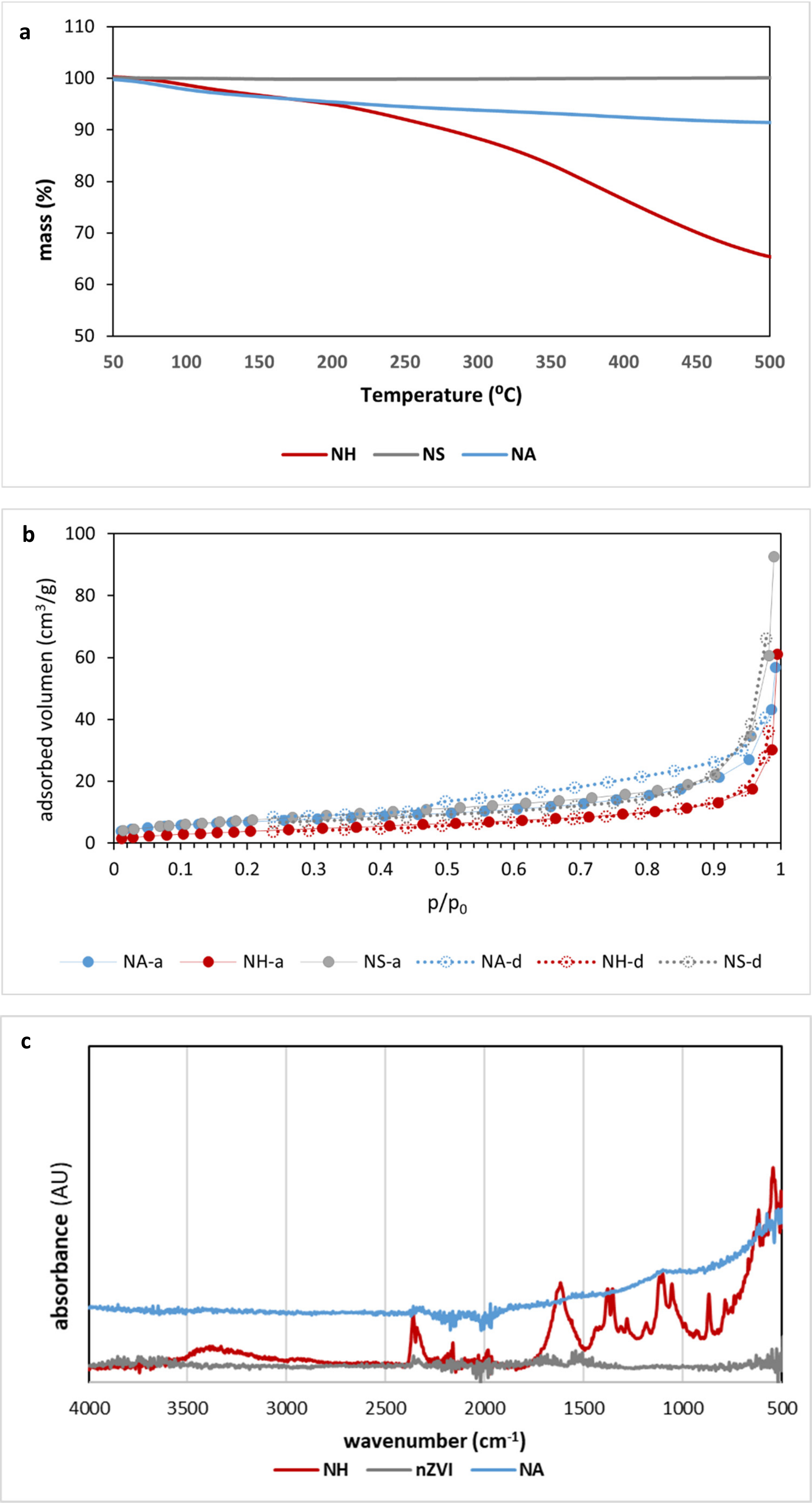
a
mass (%)
Temperature (°C)
NH
NS
NA
b
adsorbed volumen (cm3/g)
p/p0
NA-a
NH-a
NS-a
NA-d
NH-d
NS-d
c
absorbance (AU)
wavenumber (cm-1)
NH
nZVI
NA

Figure S1. Thermogravimetric analysis in N_2 for NA, NH and NS (a). Nitrogen adsorption-desorption isotherms of NA, NH and NS at 77 K (b). FTIR spectra of NA, NH and NS (c).
